# Mendelian randomization analysis reveals causal relationship between obstetric-related diseases and COVID-19

**DOI:** 10.1186/s12985-024-02348-4

**Published:** 2024-03-25

**Authors:** Yan Fang, Dajun Fang

**Affiliations:** Department of Obstetrics and Gynecology, Guangzhou Women and Children’s Medical Center, Guangzhou Medical University, No 9 Jinsui Road, Tianhe District, Guangzhou, Guangdong Province 510623 People’s Republic of China

**Keywords:** COVID-19, Obstetric-related diseases, Placental disorders, Poor outcomes

## Abstract

**Background:**

Several observational studies demonstrated that pregnant individuals with COVID-19 had a higher risk of preeclampsia and preterm birth. We aimed to determine whether women with COVID-19 diagnosis had adverse pregnancy outcomes.

**Methods:**

A two-sample Mendelian randomization (MR) analysis in this study was used to evaluate the casual relationships between COVID-19 infection and obstetric-related diseases based on genome-wide association studies (GWAS) dataset. Inverse-variance weighted (IVW), MR-Egger and MR-PRESSO were used to infer the connection and estimate the pleiotropy respectively.

**Results:**

The significant connection was observed between COVID-19 and placental disorders with beta_IVW_ of 1.57 and odds ratio (OR) of 4.81 (95% confidence interval [CI]: 1.05–22.05, *p* = 0.04). However, there were no associations between COVID-19 infection and gestational diabetes mellitus (GDM) (OR = 1.12; 95% CI: 0.85–1.45, *p* = 0.41), other disorders of amniotic fluid and membranes (OR = 0.90; 95% CI: 0.61–1.32, *p* = 0.59), Intrahepatic Cholestasis of Pregnancy (ICP) (OR = 1.42; 95% CI: 0.85–2.36, *p* = 0.18), birth weight (OR = 1.02; 95% CI: 0.99–1.05, *p* = 0.19), gestational hypertension (OR = 1.00; 95% CI: 1.00–1.00, *p* = 0.85), spontaneous miscarriages (OR = 1.00; 95% CI: 0.96–1.04, *p* = 0.90) and stillbirth (OR = 1.00; 95% CI: 0.98–1.01, *p* = 0.62).

**Conclusion:**

There was no direct causal relationship between COVID-19 infection and maternal and neonatal poor outcomes. Our study could alleviate the anxiety of pregnant women under the COVID-19 pandemic conditions partly.

**Supplementary Information:**

The online version contains supplementary material available at 10.1186/s12985-024-02348-4.

## Highlights

1. COVID-19 was positively related to placental disorders.

2. COVID-19 didn’t cause stillbirths, spontaneous miscarriages, gestational hypertension/pre-eclampsia, low birth weight, intrahepatic cholestasis of pregnancy, gestational diabetes and other disorders of amniotic fluid and membranes.

**Supplementary Information:**

The online version contains supplementary material available at 10.1186/s12985-024-02348-4.

## Introduction

The coronavirus disease 2019 (COVID-19) originated in Wuhan had killed millions of people and its pandemic led to panic worldwide and its pathogenic virus was severe acute respiratory disease coronavirus 2 (SARS-CoV-2) [1]. It was reported in 2020 that 60% newborns were born prematurely, 20% were small for gestational age (SGA) neonates and most infants had symptoms of shortness of breath in 10 newborns with negative nucleic acid born to mothers with COVID-19 [2]. The results from large multicenter cohort study showed that pregnant women with COVID-19 diagnosis had an increased risk of hypertension-related diseases, preterm birth, fetal distress, stillbirth, low birth weight and maternal deaths [3, 4]. Conversely, another retrospective study elucidated that COVID-19 couldn’t cause severe perinatal outcomes such as preterm birth nor can it be transmitted to the fetus through placenta [5], and some researchers also showed that COVID-19 pandemic had no effect on rates of spontaneous abortion through cross-sectional study [6]. In the meanwhile, to our knowledge, there were no studies to explore the specifical mechanism of COVID-19 and obstetric-related diseases. However, previous studies have pointed out that after pregnant women were infected with SARS-CoV-2, the transcription of placental syncytial trophoblast cells was changed, resulting in impaired cellular processes and reduced secretion of HCG hormone, resulting in impaired placental barrier [7]. The impaired placental barrier in pregnant women will not only cause damage to the pregnant women themselves, but also cause damage to the fetus through peroxide stress [8]. Thus, it is very essential to explore the related causes of obstetric-related diseases. And it was clear that there were conflicting results about the impact of COVID-19 on pregnancy, which might stem from economic instability and medical restrictions [9], therefore it was worthwhile for us to infer the causal relationships between COVID-19 infection and obstetric-related diseases.

Mendelian randomization (MR) was a data analysis technique to evaluate causal inference in epidemiological studies [10]. It used genetic variants as instrumental variables to assess the causal relationship between the exposure and the outcome of interest in non-experimental data [11]. “Exposure factor” referred to a putative causal risk factor, also known as an intermediate phenotype, which could be a biomarker, a physical measurement, or any risk factor that might affect outcomes [12]. Different from traditional randomized trials which were time-consuming, laborious, expensive, some not ethically supported and easy to cause bias because of behavioral, social, psychological and other factors [13], while MR analysis used single nucleotide polymorphisms as instrumental variables to make the results more reliable [14, 15]. In this study, we used MR analysis to evaluate the relationship between obstetric-related diseases and COVID-19, and inverse-variance weighted (IVW) MR analyses demonstrated that there was a statistically significant association between pregnant women diagnosed with COVID-19 infection and placental disorders, and the OR value was 4.81 (95%CI: 1.05–22.05, *P* = 0.04). Moreover, COVID-19 had null association with other traits. Furthermore, MR‐Egger regression revealed no statistically significant intercept for all traits and the *p* value in IVW test of heterogeneity analysis was greater than 0.05. In conclusion, our results showed that COVID-19 infection didn’t cause stillbirths, spontaneous miscarriages, gestational hypertension/pre-eclampsia, low birth weight, intrahepatic cholestasis of pregnancy, gestational diabetes and other disorders of amniotic fluid and membranes, but it led to placental disorders.

## Materials and methods

**Fig. 1:**
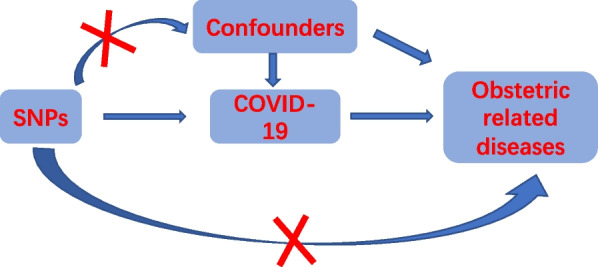
The flow chart about three key assumptions in Mendelian randomization study

We performed two-sample MR analysis with available summary-level data from the commonly available genome wide association studies (GWAS), The flow chart was shown in Fig. 1. Declaration of Helsinki statement and written informed consent had been obtained in the original publications. The summary-level data has been publicly published at https://gwas.mrcieu.ac.uk website for analysis.

### COVID-19

Genetic instruments of COVID-19 (ID: ebi-a-GCST011073) were obtained from a large-scale study including 1,683,768 participants (1,644,784 controls *vs* 38,984 cases) from European and 8,660,177 SNPs [16].

### Obstetric-related diseases

Obstetrician-related diseases refer to diseases with high incidence in obstetrics, including gestational diabetes, other disorders of amniotic fluid and membranes, Intrahepatic Cholestasis of Pregnancy (ICP), birth weight, gestational hypertension, spontaneous miscarriages, stillbirth and placental disorders [17]. The datasets we used were summary-level datasets and included populations from different European countries, therefore the diagnostic thresholds for the following common obstetrician-related diseases were not uniform. Gestational diabetes data (ID: finn-b-GEST_DIABETES) included 5687 cases and 117,892 controls, and 16,379,784 SNPs were obtained. Other disorders of amniotic fluid and membranes data (ID: finn-b-O15_AMNIOT_OTHER) included 1753 cases and 104,247 controls, and 16,379,393 SNPs were obtained. Intrahepatic Cholestasis of Pregnancy (ICP) (ID: finn-b-O15_ICP) included 940 cases and 122,639 controls, and 16,379,784 SNPs were obtained. Placental disorders (ID: finn-b-O15_PLAC_DISORD) included 102 cases and 104,247 controls, and 16,379,357 SNPs were obtained. Birth weight (ID: ukb-b-13378) included 261,932 participants, and 9,851,867 SNPs were obtained. Gestational hypertension/pre-eclampsia data (ID: ukb-b-13535) included 462,933 participants (1864 cases *vs* 461,069 controls), and 9,851,867 SNPs were obtained. Number of spontaneous miscarriages (ID: ukb-b-419) included 78,700 participants and 9,851,867 SNPs were obtained. Number of stillbirths (ID: ukb-b-6412) included 78,879 participants and 9,851,867 SNPs were obtained. All participants were of European descent.

### Statistical analysis

R packages including TwoSampleMR (*v* 0.5.6), MendelianRandomization (*v* 0.7.0), and MRPRESSO (*v* 1.0) were used in this study. Instrumental variables (IVs) were obtained according to the three assumptions of MR. In the three assumption, we set the threshold of *p*-value as 1 × 10^–5^ and the threshold of *r*^2^ to include more IVs because some of the MR methods we used are less prone to weak instrument bias [18, 19]. Firstly, we selected SNPs that were closely associated with the COVID-19 at a significance level of *p* < 1 × 10^–5^, furthermore, SNPs with linkage disequilibrium (*r*^2^ = 0.05, kb = 10,000) and IVs with weak bias (*F*-statistics < 10) were removed. Secondly, we excluded SNPs that were associated with confounding factors (*p* < 1 × 10^–5^) that related to COVID-19 and obstetric-related diseases. Finally, SNPs that were directly related to the outcomes of interest (*p* < 1 × 10^–5^) were excluded to obtain the IVs. The formula for calculating *R*^2^ and F-statistics is in the form.

While MAF is minor allele frequency, SD = SE $\times \sqrt{N}$, N and n are the sample size and *R*^2^ is a risk factor for the genotype the explanation the proportion of variability.

We used Cochran’s Q test in inverse-variance weighting (IVW) method to assess heterogeneity in the sensitivity analysis. Horizontal pleiotropy was estimated by the intercept of the MR-Egger regression and MR-pleiotropy residual sum and outlier (MR-PRESSO). We also assessed whether individual SNP had biases that independently affected the overall causal effect by leave-one-out methods. Odds ratios (OR) (*p* < 0.05) in this study was presented to evaluate the cause effects.

## Results

### SNP selection and validation

In summary, 9 IVs achieved genome-wide significance levels in gestational diabetes, stillbirths, intrahepatic cholestasis of pregnancy, placental disorders, other disorders of amniotic fluid and membranes, low birth weight and stillbirth, 7 IVs were obtained to be related to gestational hypertension/pre-eclampsia and COVID-19, 8 IVs were closely associated with spontaneous miscarriages and all F-statistics were greater than ten (Supplemental file 1).

### Casual effects of COVID-19 on obstetric-related diseases

**Fig. 2:**
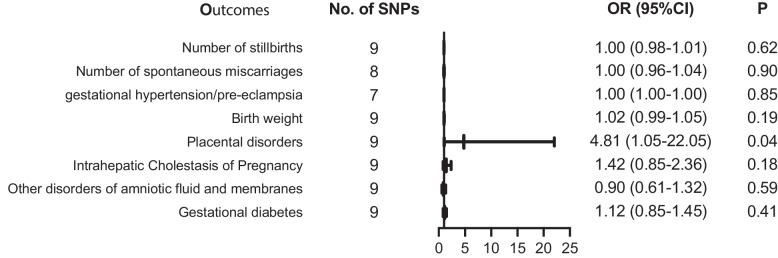
The association of genetically predicted COVID-19 with obstetric related diseases

**Fig.3:**
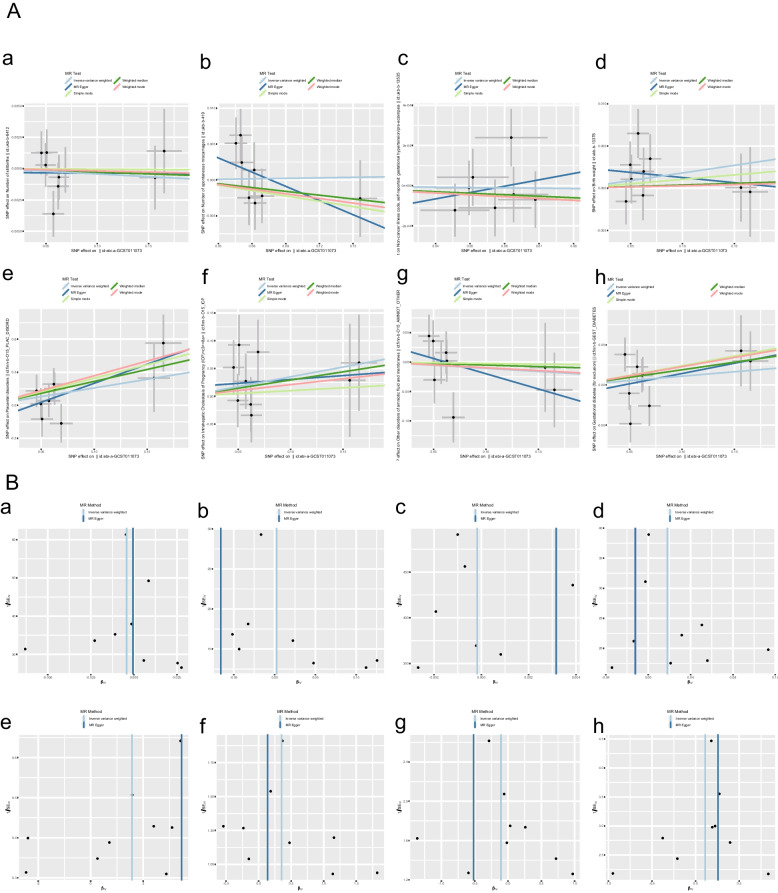
The scatter and funnel plot. **A** The scatter plot; **B** The funnel plot

**Fig. 4:**
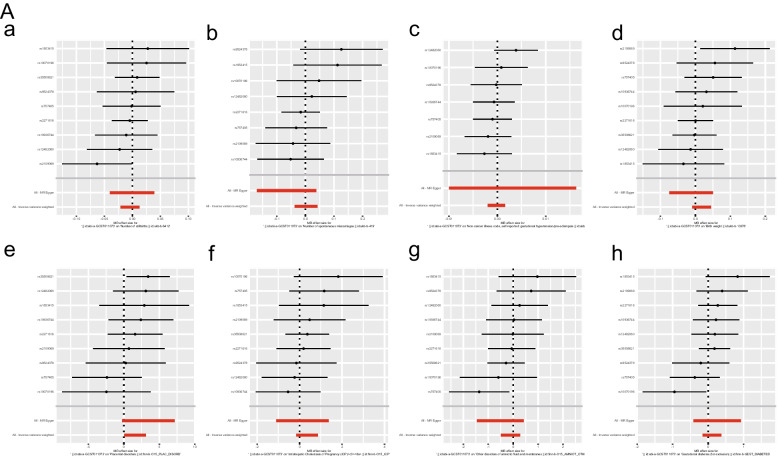
The forest plot

**Fig. 5:**
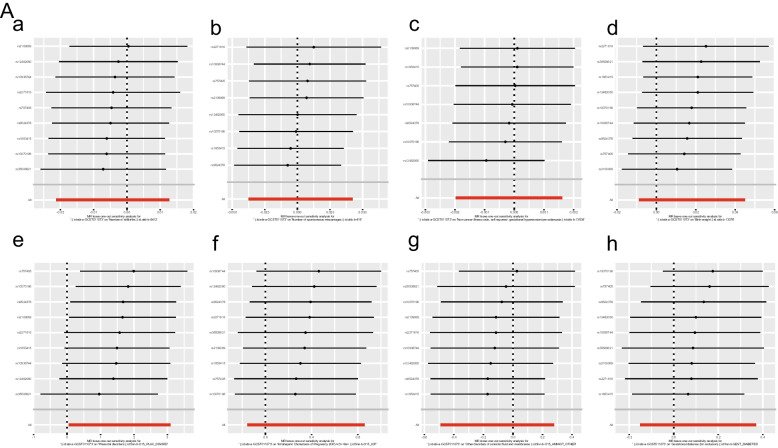
The leave-one-out plot

The IVW analysis revealed that COVID-19 infection was positively related to placental disorders with beta_IVW_ of 1.57 and OR of 4.81 (95% CI: 1.05–22.05, *p* = 0.04). However, no associations were observed for gestational diabetes mellitus (GDM) (OR = 1.12; 95% CI: 0.85–1.45, *p* = 0.41), other disorders of amniotic fluid and membranes (OR = 0.90; 95% CI: 0.61–1.32, *p* = 0.59), Intrahepatic Cholestasis of Pregnancy (ICP) (OR = 1.42; 95% CI: 0.85–2.36, *p* = 0.18), birth weight (OR = 1.02; 95% CI: 0.99–1.05, *p* = 0.19), gestational hypertension (OR = 1.00; 95% CI: 1.00–1.00, *p* = 0.85), spontaneous miscarriages (OR = 1.00; 95% CI: 0.96–1.04, *p* = 0.90) and stillbirth (OR = 1.00; 95% CI: 0.98–1.01, *p* = 0.62) (Fig. 2). For all obstetric-related diseases, MR-Egger and MR-PRESSO revealed consistent results that no evidence of horizontal pleiotropy was detected. Furthermore, the *p*-value in heterogeneity analyses was greater than 0.05 (Table 1). Scatter plot and funnel plot of the association between COVID-19 and obstetric-related diseases displayed the similar results (Fig. 3). The forest plot revealed that no horizontal pleiotropy was observed and COVID-19 was positively related to placental disorders (Fig. 4). The leave-one-out plot indicated that individual SNP didn’t affect overall estimates (Fig. 5).

**Table 1:** Heterogeneity and pleiotropy analyses

**Outcome**	**Heterogeneity**		***P* value (MR-PRESSO global test)**	**Pleiotropy**	
	**Q**	***P* value**		**Intercept**	***P* value**
Gestational diabetes	11.4473	0.1776	0.2470	-0.0113	0.6525
Other disorders of amniotic fluid and membranes	8.4574	0.3901	0.4370	0.0319	0.3790
Intrahepatic Cholestasis of Pregnancy	5.5866	0.6934	0.6090	0.0165	0.7229
Placental disorders	6.5239	0.5888	0.5850	-0.1459	0.3110
Birth weight	6.8206	0.5561	0.6090	0.0025	0.3269
gestational hypertension/pre-eclampsia	4.6963	0.5833	0.5960	-0.0002	0.6359
Number of spontaneous miscarriages	7.2041	0.4079	0.4790	0.0050	0.2118
Number of stillbirths	5.7191	0.6787	0.7320	-0.0003	0.8416

## Discussion

In our study, we used two-sample MR analysis to analyze the association between COVID-19 and obstetric-related diseases, and comprehensively assessed the causal association. Our results indicated that COVID-19 was positively correlated with placental disorders, but not with stillbirths, spontaneous miscarriages, gestational hypertension/pre-eclampsia, birth weight, intrahepatic cholestasis of pregnancy, gestational diabetes and other disorders of amniotic fluid and membranes.

A recent study documented that SARS-CoV-2 colonized the placenta cells such as syncytiotrophoblasts, extravillous trophoblasts, immune cells and cytotrophoblasts by binding to angiotensin-converting enzyme 2 receptor and transmembrane serine protease 2 (TMPRSS2) resulting in placental inflammation and malperfusion [20]. Persistent inflammatory stimuli led to the perivillous deposition of massive fibrin which affected the gas exchange between mother and fetus eventually caused the stillbirth or fetal growth restriction [21]. Other possible mechanisms were that the long-term exposure of developing fetus to intrauterine inflammation and virus resulted in adverse outcomes of obstetrics and neonatology, and in severe cases, the diseases led to multisystemic defects and death in infants [22]. Wei et al. concluded that pregnant women with COVID-19 had 1.33- fold increased risk of preeclampsia, 1.82 times higher prevalence of preterm birth and 2.11-fold risk of stillbirth, moreover, the incidence of pregnancy-related adverse outcomes raised with the severity of infection [23]. However, our study showed that COVID-19 had null association with stillbirth etc. Placenta might be a barrier to mitigate adverse outcomes and some researchers found that no specific characteristics regardless of duration and severity of COVID-19 infection by collecting 138 placentas from 131 pregnant patients, but the limitation was that this study lacked a control group [24]. Andrea G Edlow etc. enrolled 127 pregnant women and found that no evidence to support definitive vertical transmission by virtue of detecting plasma SARS-CoV-2 viral load and antibodies in maternal, umbilical cord and neonates [25]. Based on the current evidence, it was difficult to decide whether COVID-19 was an independent risk factor for preterm birth, stillbirth, abortion and ICU admission. The reason for this limitation was that pregnant women, as a special group, had been excluded in some studies, and additionally, most retrospective studies were subject to bias caused by other confounding factors [26].

Mendelian randomization (MR) used genetic variants as instrumental variable to estimate the causal relationship, and it took advantage of allele randomization and excluded SNPs associated with confounding factors to successfully avoided bias [27, 28]. We investigated the association between COVID-19 and obstetric related diseases based on Mendelian randomization, which ensured the accuracy and authenticity of our results to a certain extent, and avoided the bias caused by other social factors. However, there were still some limitations. Firstly, we relaxed the *p*-value threshold to 1 × 10^–5^ and the value of *r*^2^ threshold to 0.05 to include more IVs [18, 19], while this may lead to an inaccurate description of the causal relationship between COVID-19 and obstetrical-related diseases. Secondly, the summary-level dataset on obstetrical-related diseases did not provide a detailed definition of each disease, which contributed to the extrapolation of the results of the present study.

## Conclusion

COVID-19 had null association with stillbirths, spontaneous miscarriages, gestational hypertension/pre-eclampsia, low birth weight, intrahepatic cholestasis of pregnancy, gestational diabetes and other disorders of amniotic fluid and membranes. In the pandemic era, it was necessary to maintain regular prenatal care to stay away from panic, and our study helped to raise public awareness of COVID-19 and provided a theoretical basis for discovering the new triggers of other obstetric complications.

### Supplementary Information

**Supplementary Material 1.**

## Data Availability

The summary-level data on COVID-19 and Obstetric-related Diseases used in this study are publicly and freely available in the GWAS database (https://gwas.mrcieu.ac.uk), with ethical approval and informed consent of participants for each cohort in the GWAS.
